# Plasma Copy Number Alteration-Based Prognostic and Predictive Multi-Gene Risk Score in Metastatic Castration-Resistant Prostate Cancer

**DOI:** 10.3390/cancers14194714

**Published:** 2022-09-28

**Authors:** Jinyong Huang, Meijun Du, Alex Soupir, Liewei Wang, Winston Tan, Krishna R. Kalari, Deepak Kilari, Jong Park, Chiang-Ching Huang, Manish Kohli, Liang Wang

**Affiliations:** 1 Department of Tumor Biology, H. Lee Moffitt Cancer Center and Research Institute, Tampa, FL 33612, USA; 2 Department of the Genomic Sciences and Precision Medicine Center, Medical College of Wisconsin, Milwaukee, WI 53226, USA; 3 Department of Molecular Pharmacology and Experimental Therapeutics, Mayo Clinic, Rochester, MN 55905, USA; 4 Department of Hematology and Oncology, Mayo Clinic, Jacksonville, FL 32224, USA; 5 Department of Quantitative Health Sciences, Mayo Clinic, Rochester, MN 55905, USA; 6 Department of Oncology, Medical College of Wisconsin, Milwaukee, WI 53226, USA; 7 Department of Cancer Epidemiology, H. Lee Moffitt Cancer Center and Research Institute, Tampa, FL 33612, USA; 8 Joseph J. Zilber School of Public Health, University of Wisconsin Milwaukee, Milwaukee, WI 53205, USA; 9 Division of Oncology, Department of Medicine, University of Utah Huntsman Cancer Center, Salt Lake City, UT 84112, USA

**Keywords:** prostate cancer, cell free DNA, algorithm, prognosis, predictive biomarker

## Abstract

**Simple Summary:**

At a genomic level metastatic castrate resistant prostate cancer state is highly heterogeneous and no clear genome-based prognostic or predictive biomarkers exist in practice. We evaluated multiple copy number somatic alterations in two castrate resistant patient cohorts to determine if a genome-based risk score at the copy number level can predict clinical outcomes. The first cohort included patients in a prospective clinical-trial in which abiraterone acetate was given and the other comprised of a real-world hospital-registry. We extracted plasma cell free DNA in both cohorts and performed low pass whole genome sequencing. Copy number alterations were identified for 24 candidate genes and a final composite score developed from 11 genes. This risk score was able to predict survival in castrate resistant patients after adjusting for known clinical biomarkers. Additionally, the multi-gene copy number alteration based risk score algorithm also predicted if abiraterone acetate would be effective in castrate resistant patients.

**Abstract: Background:**

A plasma cell-free DNA (cfDNA) multi-gene copy number alteration (CNA)-based risk score was evaluated to predict clinical outcomes in metastatic castrate resistant prostate cancer (mCRPC) patients. **Methods:** Plasma specimens from two independent mCRPC patient cohorts (N = 88 and N = 92 patients) were used. A treatment-naïve mCRPC cohort (prospective clinical-trial cohort) included plasma samples before treatment with abiraterone acetate/prednisone and serially at 3-months. A separate real-world hospital-registry (RWHR) mCRPC cohort included a single blood sample collected prior to mCRPC treatments in 92 mCRPC patients following ADT failure. Low pass whole genome sequencing was performed on plasma cell-free DNA (cfDNA) and copy number alterations (CNAs) were identified for 24 candidate genes of interest. Associations of individual gene CNAs with 3 month primary resistance to therapy, progression-free survival (PFS) in the prospective trial cohort and overall survival (OS) in both cohorts was evaluated by Cox regression. A multi-gene risk score was determined for significantly associated candidate CNAs for predicting clinical outcomes. Clinical factors were included in the risk model for survival. Statistical significance for all tests was set at 0.05. **Results:** In the prospective trial cohort, patients responding to treatment were observed to have a significant copy number decrease in *AR* (*p* = 0.001) and *COL22A1* (*p* = 0.037) at 3 months, while the non-responder group showed a significant CNA decrease in *NKX3.1* (*p* = 0.027), *ZBTB16* (*p* = 0.025) and CNA increases in *PIK3CB* (*p* = 0.006). Based on the significance level of each gene, CNAs in 11 of the 24 genes (*AR, COL22A1, MYC, NCOR1, NKX3.1, NOTCH1, PIK3CA, PIK3CB, TMPRSS2, TP53, ZBTB16*) were selected to develop a Cox-regression coefficient-based weighted multi-gene risk score for predicting mCRPC outcomes in both cohorts. A higher multi-gene risk score was observed to have poor OS in mCRPC patients in the prospective trial cohort (*p* = 0.00019) and for the RWHR cohort, (*p* < 0.0001). A higher risk score was also associated with poor PFS in the prospective cohort (*p* = 0.0043). **Conclusions:** A multi-gene CNAs-based risk score derived from plasma cfDNA may predict treatment response and prognosticate survival in mCRPC and warrants prospective validation of risk-based algorithms.

## 1. Introduction

Prostate cancer (PCa) accounted for more than 34,000 deaths in US males [1] and for over 325,000 deaths world-wide [2] in 2021. The therapeutic landscape in PCa treatment has evolved rapidly and androgen deprivation therapy (ADT)—based drug combinations have emerged as preferred interventions over ADT alone in different states of progression [3]. While these regimens slow disease progression, there is inevitable progression to metastatic castrate resistant prostate cancer (mCRPC) and death [4].

Progressive states in prostate cancer from organ-confined disease to mCRPC are characterized by clonal evolution with the appearance of novel genomic instability signatures, which are either by-stander events or biological drivers of progressive disease. Novel genomic alterations have been reported in tissue [5] and plasma [6] that have been observed to be associated with clinically progressive states in the continuum of cancer progression. Prostate cancer is observed to have all types of genomic alterations but copy number alterations (CNAs) are frequent genomic events and CNAs in multiple gene loci have been observed to prognosticate and predict clinical outcomes in the mCRPC state [6,7,8,9,10,11,12,13,14]. A representative gene in mCRPC is the androgen receptor (*AR*), which is amplified in the mCRPC state and is linked to treatment resistance and poor outcomes with AR Pathway Inhibitors (ARPIs) [15,16] including abiraterone acetate/prednisone and enzalutamide [17,18]. Recently other ARPIs such as darolutamide [19] and apalutamide [20] have also been used in metastatic hormone-sensitive states, but no clear efficacy biomarkers are known for these drugs. Since at a genomic level mCRPC is a highly heterogeneous disease we previously reported a composite multi-gene CNA-based risk score in mCRPC that could be used to predict clinical outcomes. In our current study, we now enlarge the scope of using a novel multi-gene risk score calculation to observations in two independent mCRPC cohorts.

## 2. Materials and Methods

### 2.1. Study Cohorts

Plasma samples were obtained from patients enrolled in two cohorts in a single institution (Mayo Clinic). Cohort 1 included patients enrolled in a prospective trial, “**PRO**state cancer **M**edically **O**ptimized genome enhanced **T**h**E**rapy (PROMOTE)” (NCT # 01953640). Bio-specimen collections were performed prospectively across three different Mayo Clinic sites (Mayo Clinic Rochester, Mayo Clinic Florida and Mayo Clinic Arizona). The second independent mCRPC cohort consisted of patients collected in a **R**eal-**W**orld **H**ospital **R**egistry (**RWHR**) which enrolled advanced prostate cancer patients at Mayo Clinic, Rochester. The PROMOTE study had an a priori stated primary aim of determining somatic metastatic tissue-based tumor genome alterations associated with abiraterone acetate + prednisolone (AA/P) resistance at 3 months using a composite progression endpoint as per the recommendations of the Prostate Cancer Working Group-2 criteria (PCWG2) [21]. This composite endpoint data was collected prospectively and was also used in correlative bio-specimen associations. In the PROMOTE cohort, 88 chemotherapy-naïve mCRPC patients who had disease progression upon androgen-deprivation therapy (ADT) were enrolled from May 2013 to August 2015 and followed until December 2018. All patients had a baseline (pre-treatment) blood sample collection performed before initiating AA/P treatment and a second blood sample collection at 3 months of AA/P treatment (post-treatment). These blood samples were used for the current study. In the RWHR cohort, 92 mCRPC patients following ADT failure were enrolled from September 2009 to July 2013 and followed until May 2021 and had only one blood sample collected prior to mCRPC treatments which were used for the present study. All patients in these cohorts were followed until death or censored at last follow-up. This cohort was enrolled before abiraterone acetate or any novel treatments used at present for mCRPC state were in use and so no drug-based progression free survival outcome was collected. An additional 15 plasma specimens from healthy donors were collected as normal controls from the Medical College of Wisconsin. All patients in both cohorts and healthy donors provided written informed consent. These studies were approved by Institutional Review Boards at the Medical College of Wisconsin and Mayo Clinic (IRB# for the PROMOTE cohort: MC 13-001296; IRB# for the RWHR cohort: MC 09-001889).

### 2.2. cfDNA Extraction and Low-Pass Whole Genome Sequencing

Blood collection and processing in both cohorts have been previously described [10,22]. Whole blood collected in 4.5 mL Ethylenediaminetetraacetic acid (K2 or K3 EDTA) tubes from mCRPC patients and healthy donors were initially centrifuged at 2000 rpm for 10 min. The supernatant (plasma) was then fractioned into multiple aliquots for storage at −80 °C. Plasma samples underwent a second centrifugation at 3000 rpm for 10 min to make plasma poor platelet fractions before DNA extraction to ensure complete depletion of cell debris. cfDNAs were extracted from 400–800 μL of the platelet poor plasma obtained after the second spin using QIAamp DNA Blood Mini Kit (QIAGEN, Valencia, CA, USA). The final DNA eluent (50 μL) was quantified by a Qubit 2.0 Fluorometer (Life Technology, Carlsbad, CA, USA). DNA libraries were prepared using a ThruPlex DNA-Seq Kit (Rubicon Genomics, Ann Arbor, MI, USA). 12–24 libraries were pooled in a single lane for 50 bp single-end sequencing using a HiSeq2000 Sequencing System (Illumina, San Diego, CA, USA).

### 2.3. Sequencing Data Processing and Gene-Specific Copy Number Calling

Fastp (Version 0.20.1) was used for quality control of raw sequence reads with the default settings [23]. Bowtie-2 (Version 2.4.2) was then used to map the sequence reads to the human genome (hg19) with the default settings [24]. SAMtools (Version 1.11) command lines were used to convert the file format from SAM to BAM, followed by sorting, indexing, and removing duplicate reads [25]. FeatureCounts from the Subread package (Release 2.0.3) was used to call read counts for each gene [26]. The Human Release 19 comprehensive gene annotation (https://www.gencodegenes.org/human/release_19.html, accessed on 1 December 2021) (GRCh37.p13) was used as a reference.

### 2.4. Copy Number Alteration Analysis

For the current study, we chose 24 candidate genes of interest for developing a multi-gene risk score. The selection of these genes is based on our previously published multi-gene risk score initial report [10] and additionally included curated gene candidates from other studies published in the literature that reported CNA association with clinical outcomes in mCRPC state [5,27]. Details of genes with reference to previous studies have been listed in Supplementary Table S1. To make calls for the copy number status, a gene-specific log2 ratio was calculated by dividing sequence reads mapping to a gene in a patient to median sequence reads mapping to the same gene in normal controls, followed by log2 transformation. Genomic gain was defined as log2 ratio > 0.3 and genomic loss was defined as log2 ratio < −0.3. The oncoPrint function in the R package ‘ComplexHeatmap’ (Version 2.10.0) was used for the visualization of multiple genomic alteration events [28]. In the PROMOTE cohort, copy numbers changes before and after the AA/P treatment were evaluated using paired *t*-test for determining the association between plasma-based CNAs and primary resistance.

### 2.5. Survival Analysis

Prognostic and predictive clinical endpoints were evaluated for single gene CNAs and for multi-gene risk scores. For prognosis in both cohorts, overall survival (OS) was calculated from the date of study enrollment at the time of ADT failure for metastatic hormone-sensitive prostate cancer to the date of death or the date of the last follow-up for both cohorts. For the prediction of treatment outcome in the PROMOTE cohort, progression-free survival (PFS) was calculated from the date of study enrollment at the time of ADT failure to the date of AA/P treatment failure. R package ‘survival’ (Version 3.2-13) was used for the Cox proportional hazards regression analysis. R package ‘survminer’ (Version 0.4.9) was used to illustrate Kaplan–Meier survival curves.

### 2.6. Multi-Gene Risk Scores Analysis

To calculate risk scores, each gene’s CNA status (gain, loss, or no change) was first fitted into a Cox proportional hazards regression model. The weight of effect was determined using the coefficient of Cox regression results, where a positive coefficient was associated with worse clinical outcomes (OS/PFS). The composite risk score was then calculated by the following formula:

Sum [Cox regression coefficient × CNA status (1 for gain or loss, and 0 for no change) of each gene selected (ranked by best *p* values)].

The Cox proportional hazards regression model was also used to evaluate each of available clinical variables in the two independent cohorts. A combined risk score was established by combining selected multi-genes and clinical variables using the same formula described above.

Leave One Out cross-validation (LOOCV) was performed to prevent overfitting for both cohorts. Kaplan–Meier survival curves were used to illustrate associations between risk scores and OS/PFS. In the PROMOTE cohort, the changes in risk score before and after the AA/P treatment were also evaluated using paired *t*-test to characterize the pharmacodynamic effects of treatment. The associations between risk score and treatment response were tested with Fisher’s exact test. For all statistical tests involved in this study, the significance levels were set at 0.05.

## 3. Results

### 3.1. Clinical Characteristics of Study Cohorts

We enrolled 88 mCRPC patients in the PROMOTE trial of which one patient did not have specimens at baseline and five did not have plasma specimens at 3 month post-treatment. Eighty-two patients provided both pre- and post-treatment samples. The median follow-up time of this cohort is 25.85 months (cut off date for analysis November 1^st^ 2018). In the RWHR cohort, 92 mCRPC patients were prospectively enrolled at Mayo Clinic from September 2009 to January 2013 and followed until death, with a cutoff date of 13 June 2021 for analyses (median follow-up 94.67 months). The patient characteristics of the two cohorts are detailed in Table 1.

### 3.2. Broad Range of Detectable CNAs in cfDNA

**Figure 1:**
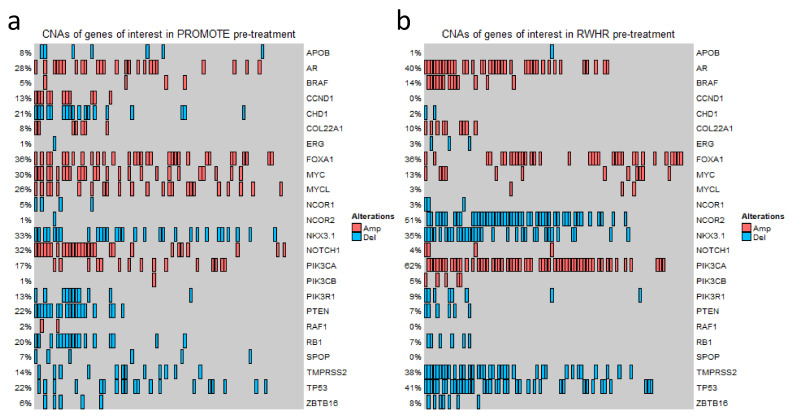
Copy number variation status of the genes of interest. OncoPrint Figure showing CNA status at 24 gene loci in the PROMOTE (**a**) and RWHR (**b**) cohorts. Each column represents an individual mCRPC patient. The percentage value indicates the CNA frequency of a gene. Red rectangle represents gain (log2 copy number ratio > 0.3). Blue rectangle represents loss (log2 copy number ratio < −0.3).

Previously, we have reported multiple genes whose copy numbers are associated with clinical outcomes of mCRPCs [5,10,13,27,29,30]. In this study, we included these candidates and then extended the list by incorporating additional genes from other publications for a total of 24 genes (Table S1). By applying the predefined cutoffs of log2 values (≥ +0.3 for gain and ≤ −0.3 for loss), a wide range of results in CNAs in different genes was observed in the two clinically similar staged independent cohorts. For example, in the PROMOTE cohort at baseline and after 3 months of AA/P treatments, we identified 36% and 40% *FOXA1* gain, 30% and 33% *MYC* gain, 28% and 12% *AR* gain, respectively. In the RWHR cohort, we detected gain in 62% patients at *PIK3CA*, 40% patients at *AR*, and 36% patients at *FOXA1*, as well as loss in 41% patients at *TP53*. Oncoprint-based figures show the detailed CNA status for each gene in the PROMOTE and RWHR cohorts (Figure 1 and Figure S1).

### 3.3. Gene-Specific and Pharmacodynamics Changes in CNAs for Primary Resistance to Abiraterone Acetate/Prednisone (AA/P) in the PROMOTE Cohort

**Figure 2:**
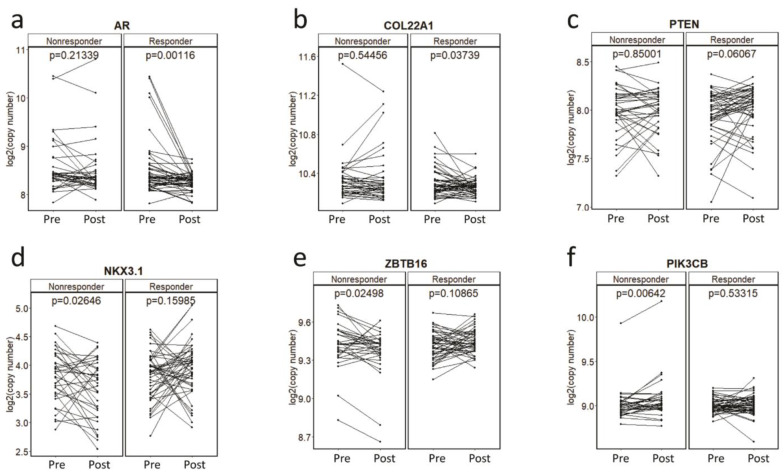
Copy number changes during the AA/P treatment. Copy number changes at *AR* (**a**), *COL22A1* (**b**), *PTEN* (**c**), *NKX3.1* (**d**), *ZBTB16* (**e**), *PIK3CB* (**f**) before (pre-) and 3-month after (post-) abiraterone acetate and prednisolone (AA/P) treatment in responders and non-responders in the PROMOTE cohort. *p* values are calculated by paired *t*-test.

To determine association of CNAs with primary resistance, we first investigated plasma CNAs in the pre-treatment PROMOTE cohort with the clinical status at 3 months of AA/P treatment failure. Patients were classified into one of the two subgroups depending on response at 3 months: as responder or progressive disease at 3 months (non-responder). The composite endpoint for PROMOTE study collected prospectively was used. This analysis did not identify any pre-treatment gene-specific CNA significant association with treatment response status at 3 months. We then evaluated pharmaco-dynamic changes of serial plasma CNAs using the pre-treatment and 3 months post-treatment. This analysis showed a significant decrease of the copy number in *AR* (*p* = 0.001) and *COL22A1* (*p* = 0.037) and a borderline significant increase of the copy numbers in *PTEN* (*p* = 0.061) in the responders at the 3 month treatment (paired t-test). However, no significant change was observed for these genes in the non-responder group at 3 months (Figure 2a–c). Instead we observed copy number decrease for *NKX3.1* (*p* = 0.026) and *ZBTB16* (*p* = 0.025), and copy number increase at *PIK3CB* (*p* = 0.006) in the non-responders to AA/P after 3 months of exposure (paired t-test) (Figure 2d–f).

### 3.4. Gene-Specific CNAs Predict Acquired Resistance and Survival

To determine gene-specific CNAs predictive of acquired resistance, we performed survival analysis using CNAs in the pre-treatment (baseline) samples of the PROMOTE cohort. We observed significant shorter PFS in patients with *AR* gain (log-rank *p* value = 0.0042, hazard ratio (HR, 95% CI) = 2.17 [1.26–3.73]), and with *COL22A1* gain (*p* = 0.022, HR = 2.47 [1.11–5.47]) (Figure S2a,b). Median PFS for patients with and without *AR* gain was 7.9 months and 13.9 months, respectively. Similarly, median PFS for patients with and without *COL22A1* gain was 5.9 months and 12.1 months, respectively. CNA status and their association with PFS are shown in Table 2. Additionally, although significant copy number changes in *PTEN* and *NKX3.1* were observed during the 3 months of AA/P treatment (Figure 2c–d), baseline *PTEN* loss and *NKX3.1* loss were not predictive of acquired resistance.

We also evaluated the prognostic value of CNAs detected in the pre-treatment samples in candidate genes in the PROMOTE cohort. Pre-treatment CNAs in the PROMOTE cohort with shorter OS were observed with gain of *AR* (log-rank *p* value = 0.018, HR = 1.98 [1.11–3.53]), *PIK3CA* (*p* = 0.022, HR = 2.16 [1.10–4.24]), and loss of *TP53* (*p* = 0.028, HR = 2.07 [1.06–4.02]), *NCOR1* (*p* = 0.0049, HR = 4 [1.41–11.40]) and *ZBTB16* (*p* = 0.00083, HR = 6.63 [1.84–23.86]) (Figure S2c–e). Table 2 summarizes CNA results of all genes along with *p*-values. The association of gene-specific CNAs with OS in the RWHR cohort was also analyzed (Table 2). Table S3 includes all CNA status raw data for each patient for all candidate genes for both cohorts.

### 3.5. Multi-Gene CNAs-Based Risk Scores Are Predictive and Prognostic in Two Separate Cohorts

mCRPC is a highly heterogeneous disease state with diverse drivers of disease progression and mechanisms of therapeutic resistance. This prompted us to go beyond a single or limited set of CNAs for determining clinical outcomes that are based on a multi-gene risk score of CNA alterations. We developed a risk score for each patient based on CNA alterations of the top 5 genes observed associated with clinical outcomes from the pre-treatment time-point (Table S2, based on Cox regression *p*-values). We then dichotomized the score at its median value for all patients in the cohort to perform survival analysis with high score (above the median) and low score (below the median) risk. To reduce sampling bias and randomness and prevent overfitting, we also performed a LOOCV to calculate the risk scores in both cohorts for the survival endpoint. The LOOCV risk scores remained prognostic of survival and predictive of acquired treatment resistance at a significant level. In the PROMOTE cohort, Kaplan–Meier survival analysis showed that a high-risk score at the pre-treatment was associated with poor OS (*p* = 0.00014) (Figure S3a). The high-risk score was also significantly associated with shorter PFS (*p* = 0.0029) (Figure S3b). In the RWHR cohort, the top 5-gene based risk score was predictive of survival in mCRPC state (*p* = 0.0054) (Figure S3c).

### 3.6. A Unified 11-Gene Risk Score Panel for Clinical Application

**Figure 3:**
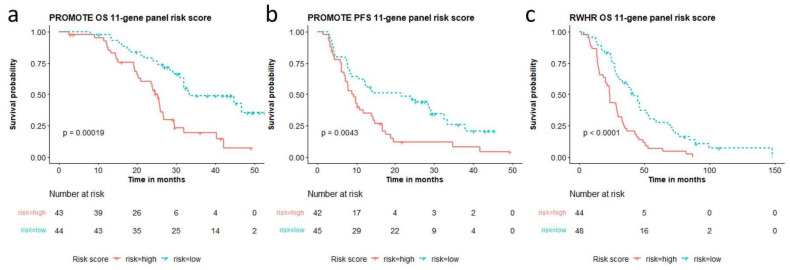
11-gene panel based high and low risk score association with overall survival (OS) and progression free survival (PFS). Risk scores calculated from the 11-gene panel (*AR*, *COL22A1*, *MYC*, *NCOR1*, *NKX3.1*, *NOTCH1*, *PIK3CA*, *PIK3CB*, *TMPRSS2*, *TP53*, *ZBTB16*) are significantly associated with (**a**) overall survival and (**b**) AA/P treatment resistance in the PROMOTE cohort, and (**c**) overall survival in the RWHR cohort.

The multi-gene risk score calculated from the top genes are cohort-dependent. Therefore, we analyzed a unified multi-gene CNA aberration score taken from the aberrations observed in both independent cohorts. We combined the top 5 genes altered for survival in both cohorts and the top five for PFS (in the PROMOTE cohort) listed in Table S2. A 11-gene panel (*AR, COL22A1, MYC, NCOR1, NKX3.1, NOTCH1, PIK3CA, PIK3CB, TMPRSS2, TP53, ZBTB16*) showed the best performance for predicting OS (*p* = 0.00019, risk score range: 0–5.42, median 0.68) as well as for predicting acquired resistance to AA/P treatment (*p* = 0.0043, risk score range: −0.44–4.01, median 0.36) in the PROMOTE cohort (Figure 3a,b). Patients with high-risk scores showed a median OS of 25.3 months and median PFS of 9.2 months, while patients with low-risk scores showed a median OS and PFS of 33.4 and 21.5 months, respectively. Notably, the risk score based on the 11-gene panel was also observed to predict survival in the RWHR cohort (*p* < 0.0001, risk score range: −0.6–7.33, median 1.07) (Figure 3c). The median OS for patients with high-risk scores was 23.2 months while with low-risk score was 41.7 months. The risk scores from the 11-gene panel remained significant after LOOCV (Figure S4a–c).

### 3.7. Multi-Gene Risk Score and Clinical Factor-Based Score Association with Outcomes

**Figure 4:**
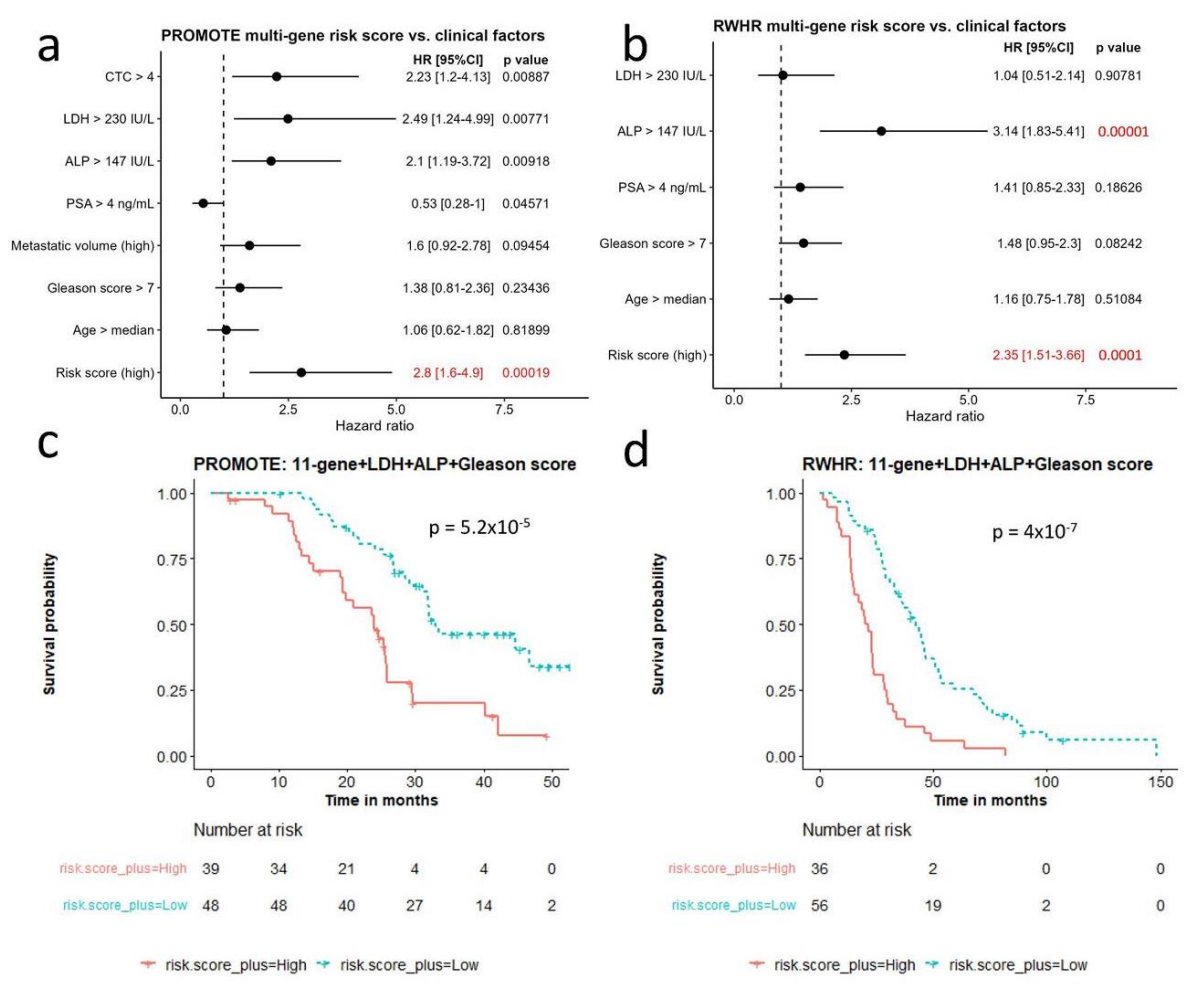
Comparison of multi-gene risk score with other clinical factors for survival. (**a**,**b**) Cox regression analysis comparing multi-gene risk score with seven survival-relevant clinical factors in the PROMOTE (**a**) and RWHR (**b**) cohort. Risk score (high) indicates 11-gene risk score above median value in the corresponding cohort. (**c**,**d**) Kaplan–Meier survival curves showing robust survival prognosis by the combination of 11-gene risk score, LDH, ALP, and Gleason score in the PROMOTE (**c**) and RWHR (**d**) cohort. CTC: circulating tumor cell; LDH: Lactate Dehydrogenase; ALP: Alkaline phosphatase; PSA: Prostate-specific antigen.

We compared the 11-gene panel with clinical factors that have been reported to be associated with castrate resistant prostate cancer survival [31,32]. We included seven known prognostic factors (circulating tumor cell (CTC) > 4, Lactate Dehydrogenase (LDH) > 230 IU/L, Alkaline phosphatase (ALP) > 147 IU/L, Prostate-specific antigen (PSA) > 4 ng/mL, high metastatic disease volume, Gleason score > 7, and Age > median age of the cohort) and performed Cox regression at the univariate level in both cohorts (Figure 4a,b). Clinical variables observed to be statistically significant at the univariate level were included with the gene risk-based score. They included serum LDH, serum alkaline phosphatase (ALP), Gleason score for survival analysis. The multivariate model performed better than univariate model alone in the PROMOTE (*p* = 5.2 × 10^−5^) (Figure 4c) and RWHR cohort (*p* = 4 × 10^−7^) (Figure 4d).

Finally, we determined pharmacodynamic changes based on the 11-gene based risk score before and after AA/P in the PROMOTE. We observed significant decrease of the risk score in responders (paired *t*-test, *p* = 7 × 10 ^−6^), while no evident changes were observed in the non-responders (*p* = 0.685) (Figure S5a). The changes in risk score during AA/P treatment (Δ risk score) were significantly correlated with treatment response (Fisher’s exact test, *p* = 0.003, odd ratio = 7.64) (Figure S5b).

## 4. Discussion

Liquid biopsies are promising tools for developing prognostic, predictive and molecular biomarkers for detection of minimal residual disease. CNAs in plasma cfDNA of mCRPC patients at specific genomic regions show strong associations with clinical outcomes (survival and treatment resistance) [5,13,27,29,30]. However, concerns remain over the applicability of these biomarkers in clinical settings because of tumor genetic heterogeneity, reproducibility and false negative results in patients with low-tumor volume. Recently it has been observed that alterations in advanced prostate cancer tissue and ctDNA show concordant cancer driver alterations making plasma ctDNA detected alterations a potentially useful tool for guiding clinical applications [33]. In this study, we targeted CNAs in 24 pre-selected genes which is a more comprehensive set of genomic variables than our previous report [10] from which the present study also differs in the approach to calculate the clinic-genomic algorithm in several ways. Even though we used two clinically matched mCRPC cohorts we observed differences in the frequencies of the candidate gene-specific CNAs. Since genetic heterogeneity in mCRPC state can make it challenging for use of a single gene-based alteration as a prognostic or predictive biomarker we explored multi-gene based cfDNA CNA-based scores to identify clinical outcomes. To accomplish this task our analytical pipeline used low pass whole-genome sequencing, gene-specific copy number calling, and then calculated a composite multi-gene risk score calculation. With this approach, we observed that the multi-gene risk score was able to predict clinical outcomes in mCRPC patients.

Several novel approaches were employed to calculate multi-gene risk scores that predict outcomes. Firstly, we used a gene-centric approach for CNA analysis. Traditionally, CNA analysis using whole-genome sequencing data is based on the sequence reads on non-overlapping genomic bins, for example, 1 Mb genomic window. In case the gene of interest is smaller than the pre-defined genomic bin, it can result in undermining gene counts. For example, *AR* and *MYC* are only around 186.6 kb and 5.4 kb in size, respectively. Therefore, the CNA signal could be significantly diluted if using an entire genomic bin to represent a gene of interest that only accounts for a small part of the bin. In addition, other genes or regulatory regions in the genomic bin may also contribute to the CNAs. To address this issue, in this study we used the sequence reads mapped to the genes of interest only for CNA analyses, which may help reduce the noise from neighboring genomic contexts and provide a more accurate gene copy change. Although this gene-centric method may result in less sequence reads in a gene locus than the genomic binning method, the reads are still adequate for CNA analysis, even for the challenging copy number loss genes such as *TP53* and *PTEN* [15].

We also observed that although CNAs were detectable in genes of interest, the frequencies of these CNAs at some gene loci were different between the two cohorts. Gain of *NOTCH1, MYCL* and loss of *PTEN, CHD1, RB1* were detected with a high frequency in the PROMOTE cohort but a lower frequency in the RWHR cohort. In contrast, loss of *NCOR2* was more frequently detected in the RWHR cohort than in the PROMOTE cohort. These findings may be attributed to selection criteria for labelling mCRPC in two different time periods (at least 5 years apart), during which time there were changing standards of care treatments for mCRPC and a tendency by clinicians to define mCRPC state earlier after 2012 than in the period between 2000–2012. Clonal evolutionary pressures may in this situation have led to differences in alteration frequencies in mCRPC patients collected in earlier or delayed mCRPC state.

Copy number gain of *AR* can drive CRPC progression while on ADT as well as during AA/P treatment [34,35]. Interestingly, we detected *AR* gain in 29% of patients in the PROMOTE cohort, but we did observe the association between *AR* gain and poor clinical outcomes during AA/P treatment. We also investigated other genes in the *AR* signaling axis. *NKX3.1* and *ZBTB16* are negative transcriptional regulators of the *AR* signaling pathway and their loss is associated with the primary resistance of abiraterone acetate and prednisone [36,37]. However, *NKX3.1* loss did not predict acquired resistance during the four-year follow-up. Although the hazard ratio of this risk factor is very high toward shorter OS, the frequency of *ZBTB16* loss was observed to be low. In addition to the *AR* signaling pathway, other pathways are also critically implicated in CRPC progression [38]. PI3K/Akt pathway is commonly altered in CRPC [39]. Consistent with previous findings, loss of the tumor suppressor gene *PTEN* and gain of *PI3K* catalytic subunit were observed in our CRPC cohorts. Both *PTEN* loss and *PIK3CB* gain were associated with the primary resistance to the AA/P treatment within 3 months. Furthermore, loss of the tumor suppressor gene *TP53* has been reported in many types of human cancers [40]. We also observed an association of *TP53* loss with shorter OS in our patient cohorts. *COL22A1* is located at 8q24.2, where the most frequent gain has been reported in primary and advanced prostate cancer. This gene encodes collagen that structurally belongs to the FACIT protein family. Our results showed a significant association of *COL22A1* gain with primary and acquired resistance toward AA/P treatment.

The other novelty of our study was to develop a multi-gene risk assessment score for clinical outcomes in two independent cohorts in mCRPC. This approach is similar to multi-gene expression panels that have been validated for clinical applications, such as the ONCOTYPE DX recurrence score used for breast cancer prognosis [41] and as a predictive biomarker [42] for deciding adjuvant chemotherapy. Identifying candidate alterations for targeted sequencing of the 11-gene panel, if validated, offers an initial step which may also be more cost effective and have higher clinical utility for practice change compared to whole genome sequencing or for determining prognostic and predictive outcomes in mCRPC state based on a single gene.

There are some limitations to this study. First, the method we used to define CNAs is novel, but exploratory, as there is no gold standard in determining gene-specific CNAs. To maintain consistency across different genes, we applied the log2 ratio of read counts in patients and read counts in healthy controls to define the CNA status of a gene locus. Since different genes have different optimal cut points for survival analysis, we decided to use 0.3 as a cut point to avoid overfitting after balancing the frequency of CNA events in both the PROMOTE and RWHR cohorts. This will need to be validated in the future using independent cohorts. Second, in the RWHR cohort, most patients have experienced different primary treatments before blood collection. Unlike in the PROMOTE cohort, samples were collected at time points when the standard of care treatments were different. This may have affected treatment-induced lineage plasticity and clonal evolutionary pressures for different results in CNAs in the two cohorts. Interestingly, despite the differences in the frequency of CNA changes between two cohorts, the 11-gene panel was able to prognosticate survival and predict treatment response. These observations are based on cohort studies and will require future validation in appropriately designed biomarker based clinical trials.

## 5. Conclusions

We developed a risk score algorithm based on cfDNA CNAs in 11 genes, which also included clinical variables specific for CRPC state. This multi-gene risk score predicts treatment resistance to AA/P in mCRPC patients and prognosticates mCRPC survival. These multiple gene CNA-based findings in cfDNA offer the potential for biomarker development that may impact future clinical management in mCRPC.

## Figures and Tables

**Table 1:** Clinical characteristics of the PROMOTE and RWHR cohort.

Patient Cohorts		PROMOTE	RWHR	*p* Value of Differences
Total		88	92	
Age				0.375
	Median	72	73	
	IQR	66–78	67–78	
	Range	39–91	43–92	
Gleason score at initial diagnosis				0.251
	2–6	16	9	
	7	27	34	
	8–10	45	44	
	Missing	0	5	
Volume of metastatic disease				<0.0001
	High	50	30	
	Low	38	62	
Site of metastases				0.901
	Bone	78	81	
	Others	10	11	
Baseline Prostate-Specific Antigen (ng/mL)				0.276
	Median	14.7	19.25	
	IQR	6.38–41.9	3.25–79.72	
Lactate Dehydrogenase (U/L)				0.4535
	Median	185.5	188	
	IQR	170.8–209.2	158.5–230.5	
	Missing	4	49	
Alkaline phosphatase (U/L)				0.767
	Median	109.5	96	
	IQR	83.25–178.75	68.25–137.50	
	Missing	6	6	
Time from ADT start to ADT fail (months)				0.224
	Median	24.87	19.97	
	IQR	11.55–53.8	7.88–44.92	
Time from date of enrollment to last follow-up (months)				0.0001
	Median	58.52	128.83	
	IQR	51.74–62.92	120.69–139.01	
Dead/alive during follow up				<0.0001
	Dead	55	86	
	Alive	33	6	
Primary radical prostatectomy				0.944
	Yes	37	42	
	No	45	50	
	Missing	6	0	

**Table 2:** Cox regression analysis results in the PROMOTE and RWHR cohort.

Gene	CNA	PROMOTE PFS		PROMOTE OS		RWHR OS	
		HR	*p* Value	HR	*p* Value	HR	*p* Value
*APOB*	Del	1.07	0.8812	0.69	0.5273	4.54	0.1061
*AR*	Amp	2.17	0.0042	1.98	0.0180	1.59	0.0377
*BRAF*	Amp	0.58	0.4452	0.38	0.3204	1.93	0.0293
*CCND1*	Amp	1.32	0.4225	1.04	0.9120	NA	1.0000
*CHD1*	Del	1.17	0.5946	1.02	0.9452	1.84	0.3899
*COL22A1*	Amp	2.47	0.0216	1.86	0.1456	3.58	0.0002
*ERG*	Del	0.00	0.3336	0.00	0.6569	0.82	0.7421
*FOXA1*	Amp	1.15	0.5805	0.78	0.3800	1.10	0.6601
*MYC*	Amp	1.57	0.0801	1.43	0.2088	0.55	0.0888
*MYCL*	Amp	0.71	0.2145	0.62	0.1294	0.89	0.8479
*NCOR1*	Del	1.98	0.1816	4.00	0.0050	7.47	0.0002
*NCOR2*	Del	0.00	0.3336	0.00	0.6569	1.53	0.0529
*NKX3.1*	Del	0.69	0.1833	1.02	0.9585	1.80	0.0084
*NOTCH1*	Amp	1.04	0.8705	1.02	0.9469	5.45	0.0005
*PIK3CA*	Amp	1.22	0.5408	2.16	0.0225	1.68	0.0247
*PIK3CB*	Amp	20.89	0.0001	3.61	0.1797	2.54	0.0391
*PIK3R1*	Del	1.13	0.7427	1.79	0.1071	1.94	0.0902
*PTEN*	Del	1.10	0.7420	1.07	0.8421	1.70	0.2116
*RAF1*	Amp	1.00	0.9964	3.06	0.1063	NA	1.0000
*RB1*	Del	1.15	0.6240	1.02	0.9515	2.49	0.0278
*SPOP*	Del	1.44	0.4378	1.32	0.5539	NA	1.0000
*TMPRSS2*	Del	0.93	0.8522	1.95	0.0965	1.73	0.0136
*TP53*	Del	1.43	0.2357	2.07	0.0286	1.50	0.0655
*ZBTB16*	Del	2.29	0.1547	6.63	0.0008	1.03	0.9405

## Data Availability

DNA sequencing data and clinical data are available upon request.

## Supplementary Materials

The following supporting information can be downloaded at: https://www.mdpi.com/article/10.3390/cancers14194714/s1, Figure S1. Copy number variation status of the genes of interest in post-treatment samples; Figure S2. Single gene CNAs predictive of acquired resistance and prognostic of survival; Figure S3. Multi-gene CNAs based risk score from the top 5 genes and clinical associations; Figure S4. Leave one out cross-validation of risk score from the 11-gene panel; Figure S5. Changes in risk scores before (pre) and 3-month after (post) the AA/P treatment in responders and non-responders in the PROMOTE cohort. Table S1. Information about the 24 candidate genes of interest; Table S2. Coefficients of top five genes from Cox regression analysis in two cohorts. Table S3: CNAs observed for all patients in both cohorts and survival times.
