# Supplementary material for: Plasma Copy Number Alteration-Based Prognostic and Predictive Multi-Gene Risk Score in Metastatic Castration-Resistant Prostate Cancer
Source: Cancers (Basel). 2022 Sep 28;14(19):4714. doi: 10.3390/cancers14194714 (PMC9562906; doi:10.3390/cancers14194714)
Supplement: Supplementary file 1 [file cancers-14-04714-s001.zip › cancers-1873511-supplementary.pdf]

Supplementary Materials: Plasma Copy Number Aberration-Based Prognostic and Predictive Multi-gene Risk Score in Metastatic Castration-Resistant Prostate Cancer

Jinyong Huang, Meijun Du, Alex Soupir, Liewei Wang, Winston Tan, Krishna R. Kalari, Deepak Kilari, Jong Park, Chiang-Ching Huang, Manish Kohli and Liang Wang

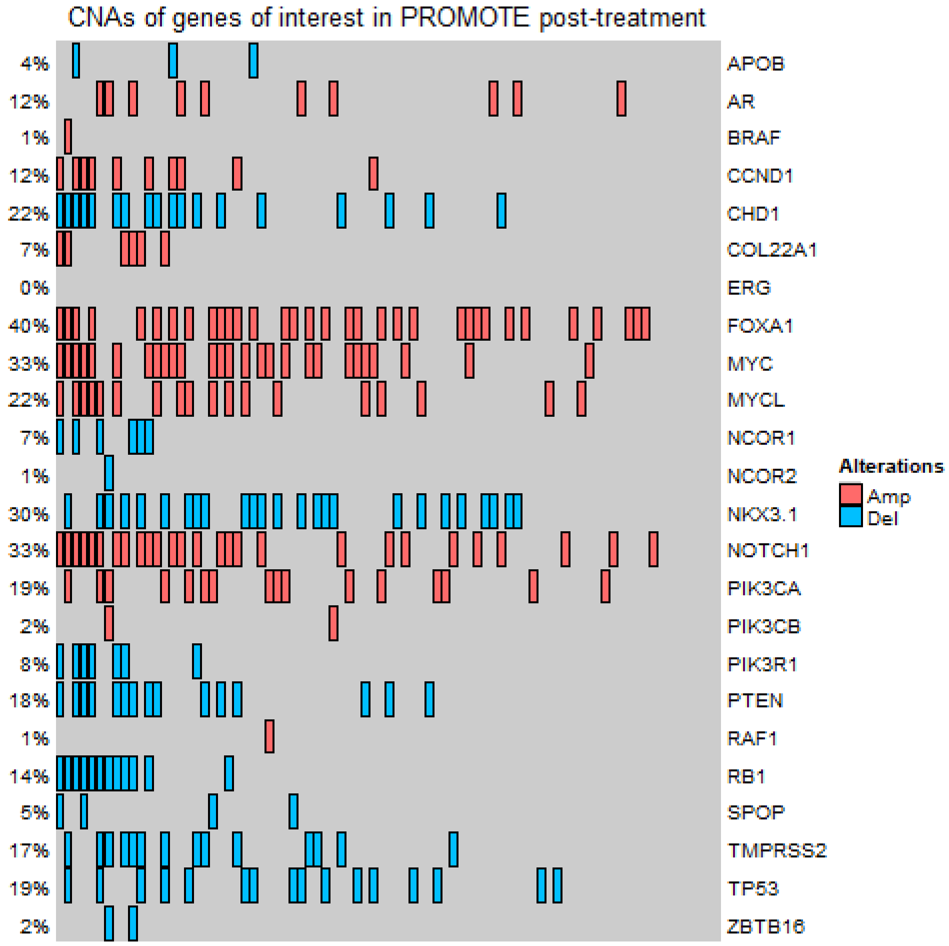

Figure S1. Copy number variation status of the genes of interest in post-treatment samples.

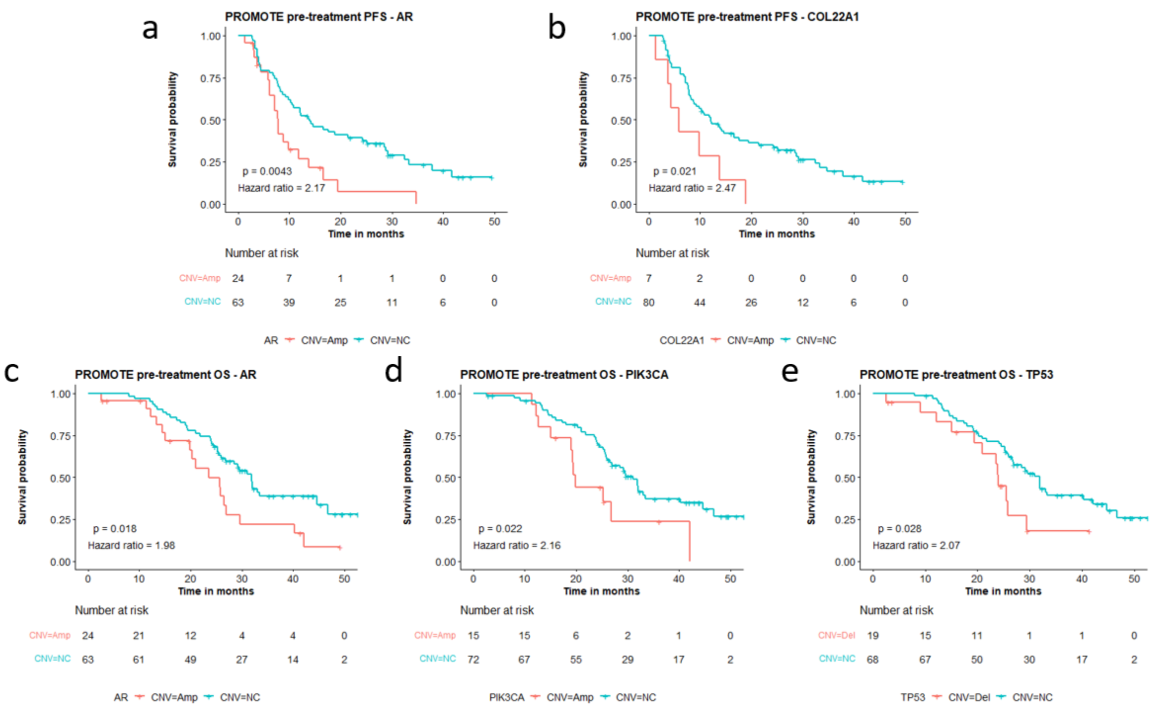

Figure S2. Single gene CNAs predictive of acquired resistance and prognostic of survival.

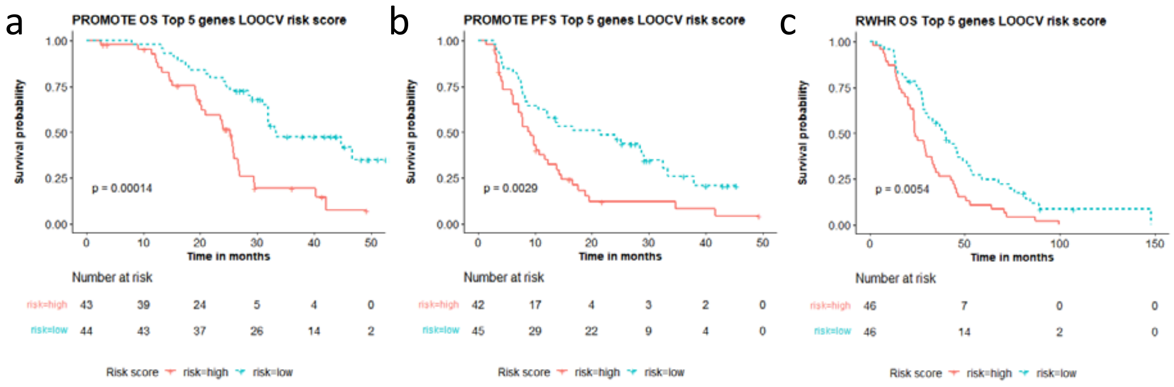

Figure S3. Multi-gene CNAs based risk score from the top 5 genes and clinical associations.

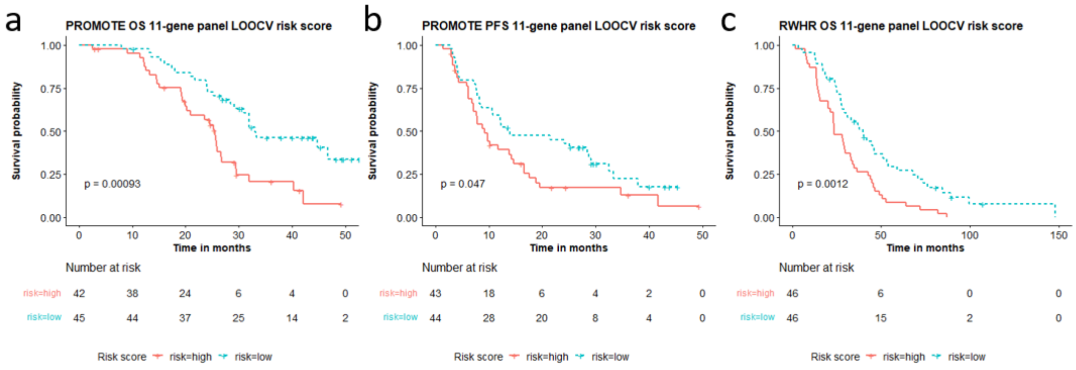

Figure S4. Leave one out cross-validation of risk score from the 11-gene panel.

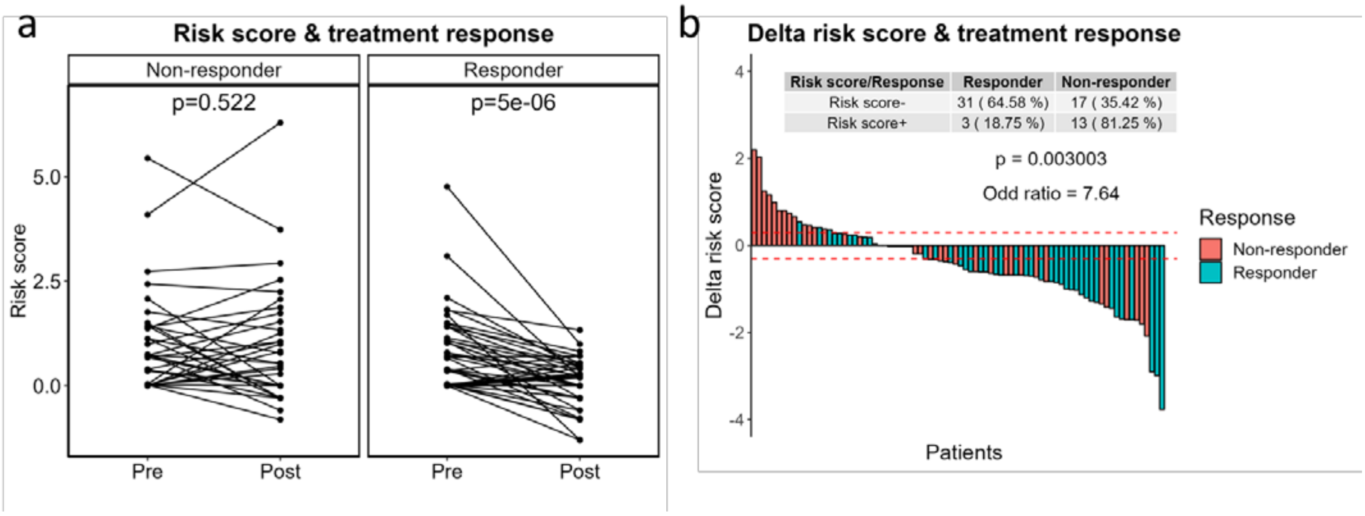

Figure S5. Changes in risk scores before (pre) and 3-month after (post) the AA/P treatment in responders and non-responders in the PROMOTE cohort.

Table S1. Information about the 24 candidate genes of interest. The previously published multigene score was based on a set of CNA aberrations in 11 genes with the endpoint of time to treatment change. Below is the list of 24 candidate genes with references chosen for the present study.

Gene	Description	GRCh38/hg38 position	Reference PMID
APOB	Apolipoprotein B	chr2:21,001,429-21,044,073	34729547, 16299398
AR	Androgen Receptor	chrX:67,544,021-67,730,619	26000489, 32203070
BRAF	B-Raf Proto-Oncogene, Serine/Threonine Kinase	chr7:140,713,328-140,924,929	26000489
CCND1	Cyclin D1	chr11:69,641,156-69,654,474	26000489
CHD1	Chromodomain Helicase DNA Binding Protein 1	chr5:98,853,985-98,929,772	26000489, 22722839
COL22A1	Collagen Type XXII Alpha 1 Chain	chr8:138,588,235-138,914,087	23636849
ERG	ETS Transcription Factor ERG	chr21:38,367,261-38,661,783	26000489
FOXA1	Forkhead Box A1	chr14:37,589,552-37,596,059	26000489
MYC	MYC Proto-Oncogene, BHLH Transcription Factor	chr8:127,735,434-127,742,951	26000489, 32203070
MYCL	MYCL Proto-Oncogene, BHLH Transcription Factor	chr1:39,895,426-39,902,256	27127882
NCOR1	Nuclear Receptor Corepressor 1	chr17:16,029,157-16,218,185	26000489, 32203070
NCOR2	Nuclear Receptor Corepressor 2	chr12:124,324,415-124,567,612	26000489, 32203070
NKX3.1	NK3 Homeobox 1	chr8:23,678,693-23,682,938	32203070
NOTCH1	Notch Receptor 1	chr9:136,494,433-136,546,048	27127882
PIK3CA	Phosphatidylinositol-4,5-Bisphosphate 3-Kinase Catalytic Subunit Alpha	chr3:179,148,114-179,240,093	26000489, 32203070
PIK3CB	Phosphatidylinositol-4,5-Bisphosphate 3-Kinase Catalytic Subunit Beta	chr3:138,652,698-138,834,928	26000489
PIK3R1	Phosphoinositide-3-Kinase Regulatory Subunit 1	chr5:68,215,737-68,301,821	26000489
PTEN	Phosphatase And Tensin Homolog	chr10:87,863,625-87,971,930	26000489, 32203070
RAF1	Raf-1 Proto-Oncogene, Serine/Threonine Kinase	chr3:12,583,601-12,664,125	26000489
RB1	RB Transcriptional Corepressor 1	chr13:48,303,744-48,599,436	26000489, 32203070
SPOP	Speckle Type BTB/POZ Protein	chr17:49,598,884-49,678,163	26000489
TMPRSS2	Transmembrane Serine Protease 2	chr21:41,464,300-41,531,116	26000489
TP53	Tumor Protein P53	chr17:7,661,779-7,687,538	26000489
ZBTB16	Zinc Finger and BTB Domain Containing 16	chr11:114,059,041-114,256,770	26000489

Table S2. Coefficients of top five genes from Cox regression analysis in two cohorts.

PROMOTE				RWHR	
Overall Survival		Progression-free Survival		Overall Survival	
**Top 5 genes**	**Coefficients**	**Top 5 genes**	**Coefficients**	**Top 5 genes**	**Coefficients**
ZBTB16	1.89	*PIK3CB*	3.04	*NCOR1*	2.01
NCOR1	1.39	*AR*	0.77	*COL22A1*	1.28
AR	0.68	*COL22A1*	0.9	*NOTCH1*	1.7
PIK3CA	0.77	*MYC*	0.45	*NKX3.1*	0.59
TP53	0.73	*ZBTB16*	0.83	*TMPRSS2*	0.55
